# Effects of Low Frequency-Low Voltage Alternating Electric Current on Apoptosis Progression in Bioelectrical Reactor Biofilm

**DOI:** 10.3389/fbioe.2020.00002

**Published:** 2020-01-22

**Authors:** Edris Hoseinzadeh, Chiang Wei, Mahdi Farzadkia, Abbas Rezaee

**Affiliations:** ^1^Department of Environmental Health Engineering, Social Determinants of Health Research Center, Saveh University of Medical Sciences, Saveh, Iran; ^2^The Experimental Forest, College of Bio-Resources and Agriculture, National Taiwan University, Taipei, Taiwan; ^3^Department of Environmental Health Engineering, School of Public Health, Iran University of Medical Sciences, Tehran, Iran; ^4^Department of Environmental Health, Faculty of Medical Sciences, Tarbiat Modares University, Tehran, Iran

**Keywords:** electrical stimulation, apoptosis, alternating current, bioelectrical reactor, flow cytometry, bioelectrochemical systems

## Abstract

Bioelectrochemical systems have undergone several modifications to promote the enzymes or pathways used to reduce the energy required for microbial metabolism. Changes in dominant bacteria, population, and growth rates occur when an electric current is applied intermittently. Applying electricity to bioelectrical reactor (BER) biofilms can either stimulate cells or lead to cell death; therefore, determining the applied voltage range that leads to viable and stimulated bacteria is crucial. We investigated the progression of apoptosis induced by a low frequency-low voltage alternating electric current (AC) in a BER biofilm and found that biofilms on carbon cloth (CC) and stainless steel (SS) 304 electrodes had pH_zpc_ values of 8.67. The pH_zpc_ of the biofilms increased by two compared to that of the inoculant bacteria mass. Furthermore, the Henderson–Hasselbalch equation reveals that the compositions of cell walls of the biofilms that formed on the CC and SS304 electrodes are very similar. In contrast, the CC and SS304 biofilms differ from the inoculant biomass without the influence of an AC field; this indicates that there are differences in the compositions of the cell walls in the present bacteria. Fourier transform infrared spectroscopy was used to compare spectra of the biofilms with that of the inoculation mass, and there were differences in shape and absorbance intensity, indicating variability in the composition, and quantity of each individual biofilm component. In addition, the dehydrogenase activity (DHA) content varied under different applied voltages; the highest DHA was obtained at 8 Vpp. A flow cytometry analysis showed a relatively low number of apoptotic cells (10.93 ± 5.19%) for the AC amplitudes studied. Thus, a low voltage-low frequency AC likely induces significant changes in bacterial metabolic activity but causes no significant change in their viability.

## Introduction

There are some configurations in bioelectrochemical systems (BESs), such as microbial electrochemical systems, where the microbial cells present contribute to electrochemical reactions that occur at the anode, cathode, or both. The microbial cells supply energy to drive the reactions and power the fuel cell used to recover energy from wastewater and microbial desalination cells (Harnisch and Schroder, 2010; ElMekawy et al., 2014; Schroder et al., 2015). Previous research on BESs have demonstrated their applicability for wastewater treatment and to treat a wide variety of pollutants (Koók et al., 2016; Jafary et al., 2017). Although using an electric current can influence the system efficiency, the resulting effects are a consideration for another study (Liu et al., 2013, 2016; Tong and He, 2013). Several recent studies have found that the low currents used to create the electric supply for BESs can stimulate changes in their surface hydrophobicity (e.g., Zeyoudi et al., 2015). Whereas, the large currents caused by electrochemical reactions taking place on the electrode surface, or elsewhere within the BES chamber, can result in the generation of oxidants or the transfer of high electric currents to bacteria. This electrical transfer could increase extracellular substances on the surface, alter cell surface charge, inactivate bacteria, reduce cell growth, or lead to cell death (Harnisch et al., 2011; Zeyoudi et al., 2015; Yang et al., 2016). To understand the parabolic dependence of biofilm cell density on an electrode when a current is applied, the surface property of the electrodes, as well as the bacteria, must be evaluated. Determining the charge types of the surfaces (i.e., the electrodes and biofilm) could directly lead to a qualitative judgment of the type of interaction (attraction or interaction) between bacteria and the surface (Hoseinzadeh et al., 2014, 2017a,b). When an electrode was maintained at a smaller potential, the biomass cells experienced a repulsion force in the initial adhesion step. The more negative the electrode potential was, the larger the repulsion force the biomass cells would experience. The initial potential of the bacteria to attach to solid surfaces has a considerable impact on the development of biofilms. To avoid adverse effects on the microbial cells, electric currents should be used in a limited range (Zhang et al., 2016). Under 10 A/m^2^, the current is low and does not affect viability, but a current density as high as 10–25 A/m^2^ results in bacterial death rates between 15 and 30% (Zeyoudi et al., 2015). When an electric current was constantly applied, no alternation in the microbial population structure occurred, and growth increased because of the electro-stimulation. In contrast, when an electric current was applied intermittently, changes in the dominant bacteria species, population, and growth rates occurred (Cheng et al., 2011; Huang et al., 2015). The application of currents >40 mA to electro-active bacteria changed the dominant microbial population and led to the generation of extracellular surface substances; however, no changes occurred at lower currents (Zeyoudi et al., 2015; Dehghani et al., 2018). Each bacterium has a different threshold for the applied current (Borole et al., 2011). When the applied electric current exceeds this threshold, cell membrane permeability will be irreversibly damaged, resulting in cell death.

Our previous studies demonstrated that the application of low voltage-low frequency alternating electric current (AC) to biofilms in an anoxic reactor led to changes in morphology, biochemical production, enzyme activity, population composition, and the generation of dense and fast-settling sludge granules (Hoseinzadeh et al., 2017b,c, 2018). In Alternating current (AC) unlike to direct current (DC) an electric current reverses direction periodically. AC has different forms, as long as the voltage and current are alternating. A waveform shows variation of alternating current (AC) with time. The most familiar AC waveform is the sine wave. Until now, several studies have been conducted concerning the use of direct current in BESs, but few have reported the use of AC. Therefore, the objective of this study was to evaluate the effect of AC on the bacterial viability of an AC bioelectrical reactor (abbreviates as ACBER). The viability and characteristics of the bacteria were assessed using several indices. The Dehydrogenase activity (DHA) and apoptosis were used for the viability assessment, while the point of zero charge (PZC), Fourier transform infrared (FTIR) spectroscopy, and cell surface hydrophobicity were used to assess the characteristics of the bacteria.

## Materials and Methods

### Bioelectrochemically Active Microbial Biofilms

All chemicals were analytical reagent grade and were used without further purification. The experiment was set up as previously described (Hoseinzadeh et al., 2017b,c, 2018). Briefly, a 5-L cylindrical glass vessel was used as the experimental bioreactor. Two electrodes, an outer electrode made from carbon cloth (CC) and an inner electrode made from mesh T304 stainless steel (SS), with an interelectrode distance of 2 cm, were mounted to the wall of the bioreactor. The CC electrode module had a mesh plastic supporting. The characteristics of the CC used were as follows: Torayca-T300B-3000-40B, 205 ± 5% g/m^2^, 103 kg/m^3^, tensile strength = 3,950 MPa, tensile modulus = 235 GPa. In addition, the used SS304 had an opening area of 52%, weight of 2.1 kg/m^2^, and thickness of 1.168 mm. Potassium nitrate, ibuprofen, KH_2_PO_4_, and K_2_HPO_4_ were used as microbial nutrients with a C:N:P ratio of 3:1:0.2. An AFG-2000 function generator (GW INSTEK; 0–10 Vpp, 0.056 A, 50 Ω) was used to supply the AC (in terms of Vpp). The AC magnitude in studied system were 1–10 Vpp, 10–60 Hz and sin., square and ramp as the applied voltages, frequency, and waveforms, respectively. Sludge from the return line of a municipal wastewater treatment plant that used the activated sludge process was used as the biomass seed to start the bioelectric reactor (BER). For further information regarding the reactor startup, please refer to Hoseinzadeh et al. (2017b,c, 2018).

### Zero Point of Charge (PZC) and Henderson-Hasselbalch

The PZC values of the SS304 mesh and CC electrodes, and the biofilm attached on each (after suspension by vortex for 15 min), were determined using the pH drift method (Hoseinzadeh et al., 2017d). To obtain PZC values of the raw electrodes they were fragmented into 1 cm pieces. In addition, in order to obtain PZC values of the biofilms that formed on the electrode surfaces they were removed 15–20 cm from the bottom of the system whereas the inoculant biomass without the influence of an AC field took as the control. The two biomasses and fragmented electrodes washed with sterilized 0.85% saline solution three times. For each one, a series of solutions with pH in the range of 2–12 were prepared using a solution of 0.01 M KNO_3_ that had been boiled to remove dissolved CO_2_ and thereafter cooled to room temperature (25 ± 2°C); 0.1 M HCl or 0.1 M NaOH was used to adjust the initial pH. Next, the samples were immersed in 20 mL of solution at each pH, shaken, and maintained at a constant temperature (25 ± 0.1°C) (for the electrodes: 1 g per 20 mL of solution). The drift in the pH (final pH) was measured after 48 h. Then the difference between the initial and final pH values (ΔpH) of the solutions were plotted against their initial pH values. The pH_ZPC_ corresponds to the pH where ΔpH = 0. All measurements were carried out in triplicate. Further information regarding the cell wall composition was obtained by analyzing the titration curves using the Henderson-Hasselbalch equation:

(1) $$\begin{array}{l} \text{pH}=\text{p}{\text{K}}_{\text{a}}+{\text{log}}_{10}\left(\frac{\left[A^{-}\right]}{\left[HA\right]}\right) \end{array}$$

Here, [HA] and [*A*^−^] are the dissociated and un-dissociated acid forms, respectively. KNO_3_ has a pKa of −1.4.

### Infrared Spectrum and Scanning Electron Microscopy

The FTIR transmission spectra were acquired in the range of 4,000–400 cm^−1^ using an FTIR spectrometer. The sampled biofilms and initial inoculation mass were washed with 0.9% NaCl without any fixation step before FTIR scanning. All samples were previously dried for 24 h at 30°C to avoid interference from water-related bands. We used a simple fixation method for biomass (biofilm and the inoculant biomass without the influence of an AC field) before SEM. Briefly, the biofilm was washed in 0.9% NaCl, fixed in 2.5% glutaraldehyde, and to remove bonded water they passed through a graded ethanol series (30, 50, 75, 90, and 100%) for 15–20 min. The scanning electron microscopy (SEM) located at the Razi Metallurgical Research Center, Tehran, Iran, was used for the biofilm morphology analysis.

### Cell Surface Hydrophobicity

The cell surface hydrophobicity was determined by measuring the bacterial attachment to hydrocarbons, as described by Gannon et al. (1991) and modified by Sanin et al. (2003). *n*-Octane was used as the hydrocarbon phase for this test. The test tubes were washed in acid and rinsed prior to use. A bacterial suspension of 3 mL was transferred to a 10 mm round-bottomed test tube. After the initial turbidity was determined with a spectrophotometer, 0.3 mL of *n*-octane was added. The mixture was vortexed for 2 min and thereafter allowed to settle for 15 min at room temperature. The final optical density of the octane-free bacterial suspension was determined. The results are expressed as percentages, calculated using the following relationship:

(2) $$\begin{array}{l} \text{Percentage hydrophobicity}\left(\%\right)=100\times \frac{\left(1-OD_{final}\right)}{OD_{initial}} \end{array}$$

where *OD*_*initial*_ and *OD*_*final*_ are the initial and final optical density, respectively.

### Michaelis-Menten Model and Biokinetics Process

The linearized Michaelis-Menten model was used, as follows:

(3) $$\begin{array}{l} \frac{1}{V}=\frac{K_{s}}{V_{\max}}\times \frac{1}{S}+\frac{1}{V_{\max}} \end{array}$$

where *V* is the substrate removal rate (g/h), *V*_*max*_ is the maximum substrate consumed (g/L·h), and *S* is the substrate concentration (g/L). The biokinetics were studied by fitting the experimental data into a modified Monod model:

(4) $$\begin{array}{l} \mu =\left(\frac{\mu_{m}P_{s}}{P_{s}+K_{s}}\right) \end{array}$$

(5) $$\begin{array}{l} \text{ln}\frac{X}{X_{0}}=\mu t\text{ or }X=X_{0}\text{ EXP }\left(\mu t\right) \end{array}$$

(6) $$\begin{array}{l} t_{d}=\frac{\ln\;2}{\mu }=\frac{0.693}{\mu } \end{array}$$

where μ, μ_*m*_, *P*_*s*_, *K*_*s*_, *X, X*_0_, *t*, and *t*_*d*_ are the growth rate, maximum growth rate, substrate, half-saturation constant, biomass concentration, biomass concentration at 0 s, time, and doubling time, respectively.

### Dehydrogenase Activity (DHA)

The dehydrogenase activity (DHA) used triphenyl tetrazolium chloride (TTC) salt as the hydrogen acceptor to compare the activity of the biomass to the electrodes (Quilchano and Marañón, 2002). To present the DHA activities, we used triphenylformazan, which is the reduced form of TTC.

### Apoptosis Assay by Flow Cytometry Analysis

Annexin V-fluorescein isothiocyanate (FITC) and propidium iodide (PI) stains were used to determine the percentage of cells within the biofilm undergoing apoptosis and necrosis on the electrodes during the bioelectrochemical denitrification operation. An apoptosis assay was conducted using the protocol supplied by the manufacturer BioVision, Inc. The cells were gently removed from the electrodes for a brief time, re-suspended with 500 μL of 1× binding buffer, and then treated with 5 μL of Annexin V-FITC and 5 μL PI. Immediately after a 5-min incubation in the dark at room temperature, each sample was analyzed using a FACSCalibur™ flow cytometer with the supplied software as the instrument. The Annexin V-FITC binding (*Ex* = 488 nm; *Em* = 530 nm) was analyzed using a FITC signal detector, and PI staining was analyzed by a phycoerythrin emission signal detector. A cytogram analysis was performed using the FLOW software version 2.4.1; the unstained cells were classified as “live,” “Left Bottom,” or “Q1 area.” Meanwhile, cells stained with Annexin V only were classified as “early apoptotic,” “Left Top,” or “Q2 area”; cells stained by both Annexin V and PI were classified as “late apoptotic,” “Right Top,” or “Q3”; cells stained by PI only were classified as “dead,” “Right Bottom,” or “Q4” cells.

## Results and Discussion

### Biofilm Characteristics by pH_ZPC_, FTIR, Henderson-Hasselbalch Equation, and Hydrophobicity

#### pH_ZPC_ of Biofilm and Electrodes

**Figure 1:**
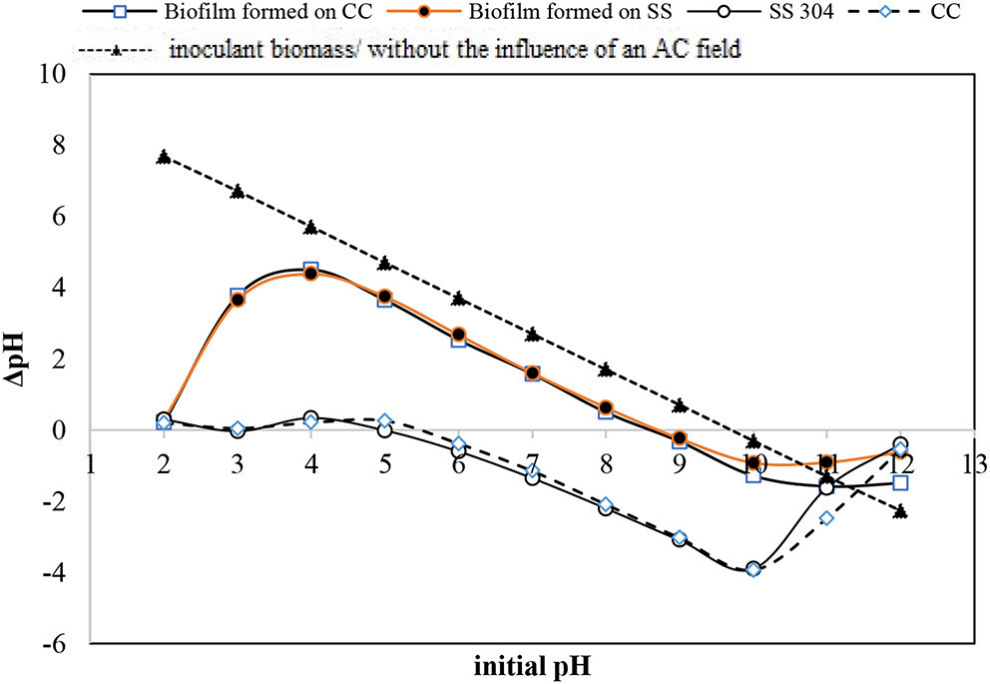
pH equilibrium [point of zero charge (PZC) measurement] for the inoculation bacteria, carbon cloth, stainless steel mesh, and formed biofilm on both.

The charges present on a bacteria cell wall are negative and cause electrostatic forces to develop in response to solid surface charges (Villanueva et al., 2014). Measurement of the pH_zpc_ is an indirect method to estimate the dominant charge type in the operating condition, particularly in aqueous solutions. The pH_zpc_ values of the CC and SS304 electrodes in this study were 5 and 5.5, respectively. Therefore, the electrode surface carried a positive charge when the bioreactor was maintained above pH 5.5, while a negative charge would be dominant when the pH was below 5.5. Biofilm development on the electrodes at different working pH values could then be perceived in terms of surface charge interaction (Hoseinzadeh et al., 2014, 2017a,b). As the pH_zpc_ of the inoculant biomass was 6.7, it can be concluded that the bacteria cells experienced a repulsion force during the initial adhesion step. Consequently, the biofilm cell density would decrease and the suspended cells in the effluent would increase (Villanueva et al., 2014). In the present study, the initial pH was adjusted to 7, changing during operation to ±0.5. We showed that the suspended biomass had an effluent turbidity of <10 NTU. This confirms that the pH can affect biofilm attachment or detachment from the surface of the electrodes. Notably, the strong attraction between bacteria and the surface may impede cell elongation and division. Bacteria do not favor proliferation on highly positively charged surfaces. Therefore, the observed cell density of the biofilm grown at varying pH values is attributed to the dual effect of the surface potential on biofilm development. The primary effect is the electrostatic interaction that dictates the initial adhesion (Villanueva et al., 2014). While the secondary effect is the binding force that inhibits cell elongation and division in the subsequent proliferation steps (Hoseinzadeh et al., 2014). The pH_zpc_ values for the biofilms on the CC and SS304 electrodes were equal, i.e., 8.67 (Figure 1). The pH_zpc_ for the biofilms increased by 2 compared with the inoculation bacteria mass. Sfaelou et al. (2015) obtained pH_zpc_ = 6.4 for biofilms formed on a porous polyvinyl alcohol gel biocarrier and a high-density polyethylene (PE) biocarrier. These data are in accordance with the pH_zpc_ of the inoculation mass in the present study. The inherent chemistry of the present system may influence the surface charge on both electrodes and biofilms, resulting from adsorbing ion(s), the development of biofilm complexities, reduced hydration in the presence of substances such as an organic carbon source (e.g., ibuprofen), and inner electro-osmotic flow within the biofilm. The change in the pH_zpc_ of the inoculate bacteria, compared to the formed biofilm, may be related to phosphorylation within the bacteria due to the applied AC (Kobir et al., 2011). Furthermore, the amount of phosphorylated residue can alter the polypeptide positions in immobilized pH gradients, which can result in an isoelectric point change. Phosphorylation is arguably the most extensively studied post-translational modification in bacteria (Kobir et al., 2011), but other alterations can occur, such as N-glycosylation, O-glycosylation, methylation, and acetylation. It can be assumed that under the influence of an AC, the charge of cell components such as phospholipids will change; therefore, forcing the whole cell charge to change. These statements are our assumptions and should be examined in detail in a future study. For bacteria in normal conditions, as the dominant anionic group, the cell wall has a negative charge, which leads to isoelectric points with a pH < 4. Furthermore, the thickness of the bacteria cell wall is influenced by the net surface charge. Isoelectric point values higher than 3 can reveal the mixed contributions of -protein or peptidoglycan-associated –COO^−^ or ${–\text{NH}}_{3}^{+}$. In addition, the combination of polymers in the cell wall and low-pKa anionic polysaccharides containing phosphate and/or carboxyl groups can result in an isoelectric point >3.

#### Henderson–Hasselbalch Equation

**Figure 2:**
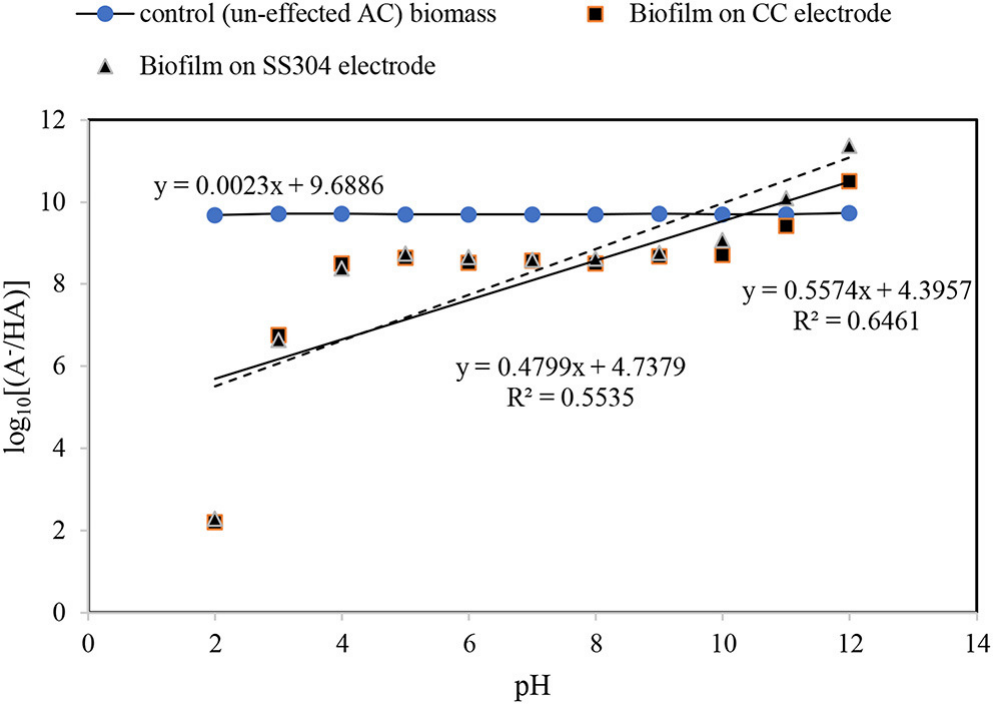
Henderson-Hasselbalch plots for the KNO_3_ and pH effect in de-attached biofilm.

The Henderson–Hasselbalch analysis (Battisti et al., 2012; Li et al., 2014) for the obtained biofilm pH_zpc_ indicates that the plot of log[*A*/*HA*] as a function of the pH results in a straight line with slopes of approximate zero (0.0023) for the inoculant biomass, without the influence of an AC field, 0.47 and 0.55 for the biofilms on the SS304 and CC electrodes, respectively (Figure 2). The Henderson–Hasselbalch curve has two points that can be identified easily and called the equivalence point (the steepest part of the curve) and where pH = PK (have the minimum slope). As Figure 2 shows this pattern curve there is not for the inoculant biomass, without the influence of an AC field. To understand relation between log[A/HA] and pH the linear relation drawn. As the results illustrate the log[A/HA] does not depend on pH for the inoculant biomass, without the influence of an AC field. For the biofilms on the SS304 and CC electrodes there is a pH (4–10) interval in which the log[A/HA] does not depend on pH and the intervals at low pH (<4) and high pH (>10) where the log[A/HA] depends strongly on pH that is in accordance with a standard Henderson–Hasselbalch curve. The inflection point of the curve shows the pHzpc of the biofilms on the SS304 and CC electrodes. The overlapped biofilm plots, taken from the biofilm formed on the CC and SS304 electrodes in the ACBER, reveal that their cell wall compositions are very similar. In contrast, the plots of CC and SS304 biofilm differ from that of the inoculant biomass, without the influence of an AC field, indicating differences in the compositions of the bacterial cell walls. The present organic cell surface groups that enable H^+^ exchange can be categorized as –COOH, –OH, and –NH_2_ (Battisti et al., 2012). This can result in alterations of the dominant surface charge, or the formation of sites comprising dominant charges. From the Henderson–Hasselbalch equation, at pH = pKa, 50% of the weak acid is ionized and when pH < pKa, ionization occurs in the logarithmic scale. However, when pH > pKa, there is a logarithmic increase in ionization. The Henderson–Hasselbalch equation is recommended for understanding the protonation of different biomolecule functional group solutions buffered to other pH levels. For carboxyl groups $\left(\frac{\left[A^{-}\right]}{\left[HA\right]}\right)=\left(\frac{\left[RCOO^{-}\right]}{\left[RCOOH\right]}\right)$ and for amino groups the ratio is $\left(\frac{\left[RNH_{3}\right]}{\left[RNH_{3}^{-}\right]}\right)$.

#### FTIR Spectra Analysis of Biofilms

**Figure 3:**
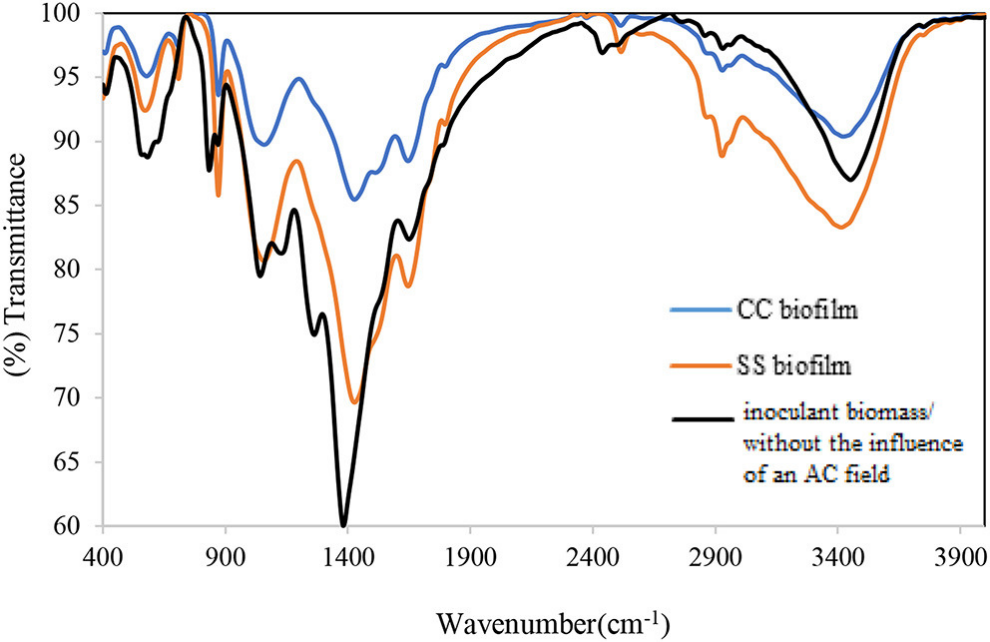
Bands of FTIR analysis of inoculation mass used in the system and formed biofilm on 12 Mesh T304 Stainless (SS 304) and carbon cloth (CC) electrodes.

**Figure 4:**
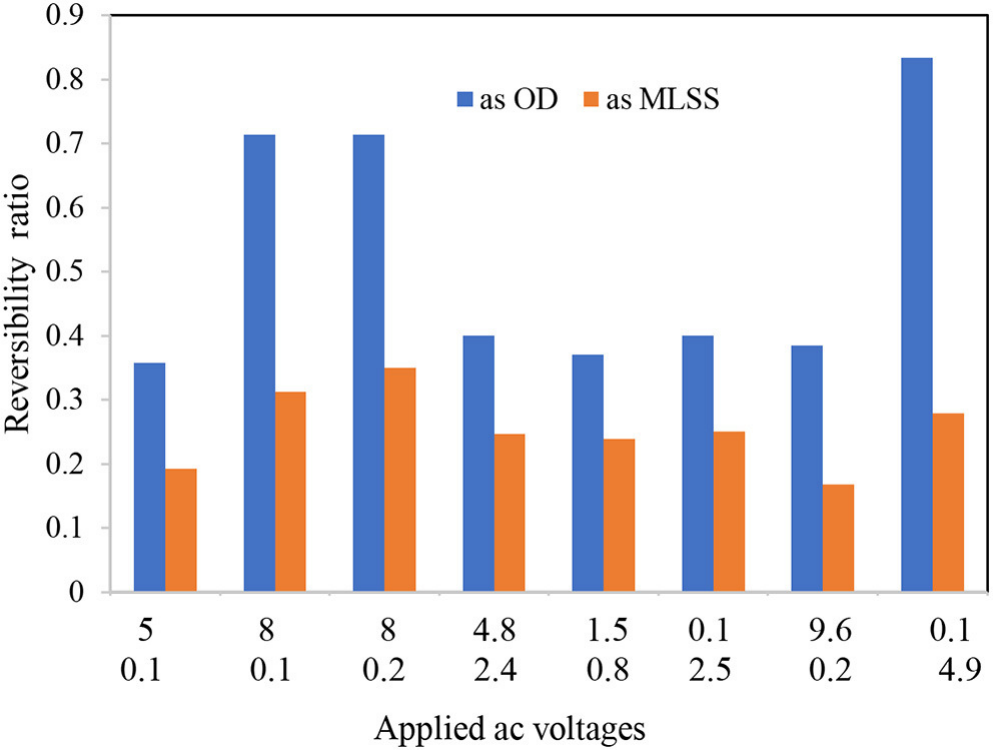
Reversibility ratio of applied AC voltages (AMPL[Vpp] and OFST[V]) to formed biofilms on the electrodes.

**Figure 5:**
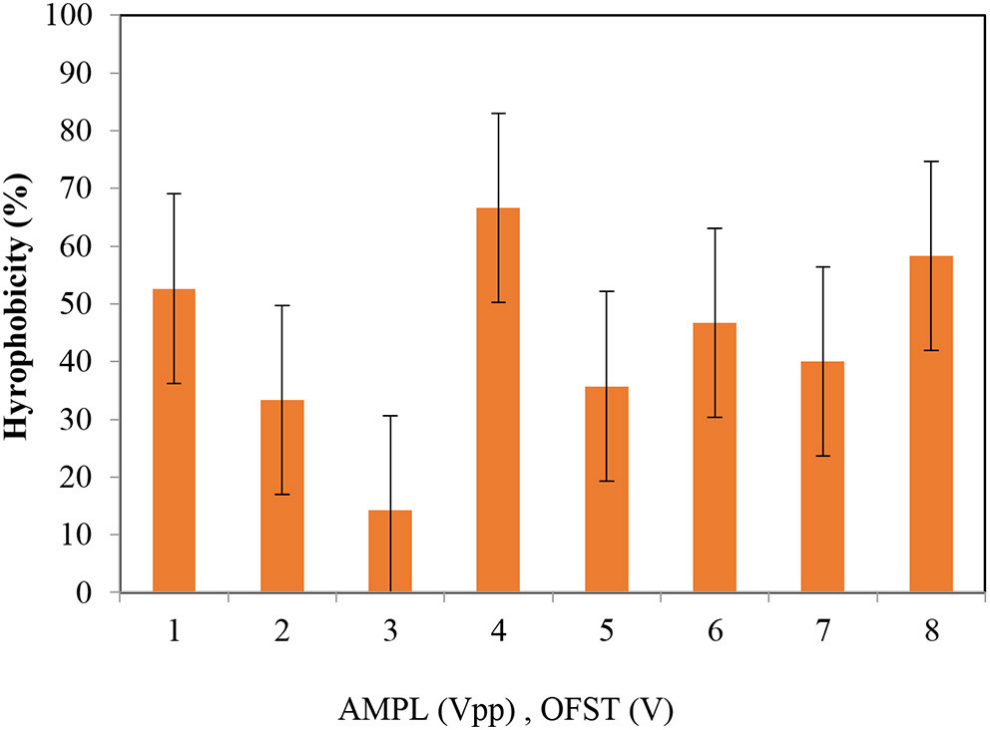
The effect of applied AC voltages on hydrophobicity of the biofilm bacteria.

The FTIR spectra analysis of the biofilms indicates the presence of polysaccharides, nucleic acids (900–1,300 cm^−1^), and proteins (1,700–1,500 cm^−1^) (Figure 3 and Table 1). Comparing the spectra of the biofilms to the inoculation mass reveals differences in both the shape and absorbance intensity, indicating a variation in the composition and quantity of each individual biofilm component. Additionally, an analysis of dried biofilm mass revealed low lipid levels (2,930–2,860 cm^−1^), whereas no lipid peaks were found in the inoculation mass (Bosch et al., 2006; You et al., 2015). The lack of variation in the protein region peaks suggests that the bacterial biological system was undamaged. Additionally, variations in the fatty acid-region peak could indicate a biological change. Proteins (amide I, II, and III bands), nucleic acids (regions around 1,240 and 1,080 cm^−1^), polysaccharides (from 1,150 to 900 cm^−1^), and lipids (around 2,900 cm^−1^) are the main groups of biomolecules (Bosch et al., 2006; Liaqat, 2009; You et al., 2015) that can be associated with the observed bands. The strong band at 1,646 cm^−1^ observed in the biofilms may partially be the result of the H–O–H bending (ν2 mode) band of water. However, other organic compounds including amide I may also have infrared bands in this region (Bosch et al., 2006). The peaks in the region of the carbohydrate C–O–C ring absorption, uronic acids, and fatty acids can confirm the presence of extracellular polymeric substances (EPSs) (Bosch et al., 2006). As the regions between 1,200 and 900 cm^−1^ are mainly correlated with the formation of EPSs, this indicates that the physical and chemical properties of the EPS may have changed more than expected during biofilm growth and alteration. Analyzing the inorganic compounds in biofilms can interfere with the corresponding biofilm spectra. For example, interferences in the carbohydrate region band can be caused by silicates, carbonates, phosphates, and iron deposits (You et al., 2015). The wavenumber of 3,400 cm^−1^, which decreases because of drying from heat, represents adsorbed water bound to the hematite (Figure 3). The bands at 3,695, 1,622, 1,520, 1,124, 1,032, 914, 600, and 475 cm^−1^ can be used to distinguish hematite from biological structures. A small organic component is indicated by the bands for the –CH_3_ and –CH groups (2,960, 2,869, 2,930, and 2,849 cm^−1^), which are evident in our FTIR analysis results of the fatty-acid region (Bosch et al., 2006; Liaqat, 2009). As Figure 4 shows the attached biofilm on the electrodes are irreversible. To find reversibility ratio de-attached cell based on turbidity and MLSS measured. The de-attached cells was measured periodically (MLSS or turbidity at initial and after of each set). Adhesion appears to be generally irreversible for all voltages applied (Figures 4, 5). This finding is consistent with previous studies that have shown adhesion to be irreversible when only weak shear forces are applied to the biofilm systems (Gall et al., 2013). In view of this, the effluent from the studied system does not need to be clarified. In addition, the biofilm formed on the electrodes is compact and electricity can easily transfer from the electrodes to the biofilm via direct electron transfer.

**Table 1:** IR assignments of formed biofilm vs. control biomass.

**Wavenumber (cm**^****−1****^**)**			**Assignments**
**Control biomass**	**Biofilm formed on CC electrode**	**Biofilm formed on SS304 electrode**	
3452.05	3422.72	3414.3	OH of water, O–H str of hydroxyl groups, and N–H str modes
–	2928.95	2927.8	**Fatty acid region:** C–H vibrations of –CH3 and >CH2 functional groups dominated by fatty acid chains (e.g., phospholipids); here, there is CH_2_ asym, stretch
2439.51	2513.7	2515.36	We found no assignment for this data
1650.31	1646.12	1646.29	**Protein region:** Amide I, (C = O) different conformation; >C=O str and C–N bending of protein and peptides amide
1382.76	1427.74	1427.86	For biofilms located in **Protein region**; it may be >C=O str (sym) of COO– and C–O bend from COO–. For inoculation mass located in **mixed region**; it may be COO– sym str.
1263.14	1058.76	1057.38	For inoculation mass located in **mixed region**; it may be COO– symstr, while for biofilms located in **Polysaccharides region**; it may be C–OH str modes and C–O–C, C–O ring vibrations of carbohydrates (oligo, polysaccharides, and alginate), C–O–P, P–O–P in polysaccharidesof cell wall. P = O str (sym) of PO_2_- in nucleic acids. This assignment is true for 1042.98 cm^−1^ of inoculation mass in next row.
1042.98 and 835.56	874.52	871.91	For biofilms only, Glycosidic linkage type “anomeric region”
–	708.08	708.71	For biofilms only, C–H rocking of >CH2 methylene
582.4	578.95	573.25	

#### Cell Hydrophobicity

The highest and lowest hydrophobicity values were obtained at the applied voltages of 4.2 and 8 Vpp, respectively (66.67 vs. 14.28%). As the hydrophobicity values change, the cell viability will also change; therefore, monitoring changes in the hydrophobicity can provide valuable information on the effect of electricity on bacteria in BESs (Van Eerten-Jansen et al., 2013). In another view, by the surface hydrophobicity results can discuss the facility of bacterial contact and bacterial adhesion to the electrodes (Hoseinzadeh et al., 2017b). If the hydrophobicity be high the bacteria attaching to the electrodes and biofilm formation enhances.

### Determining Bacterial Viability via the Michaelis–Menton Model, DHA, and Apoptosis Assay

#### Michaelis-Menten Model and Biokinetics Process

**Figure 6:**
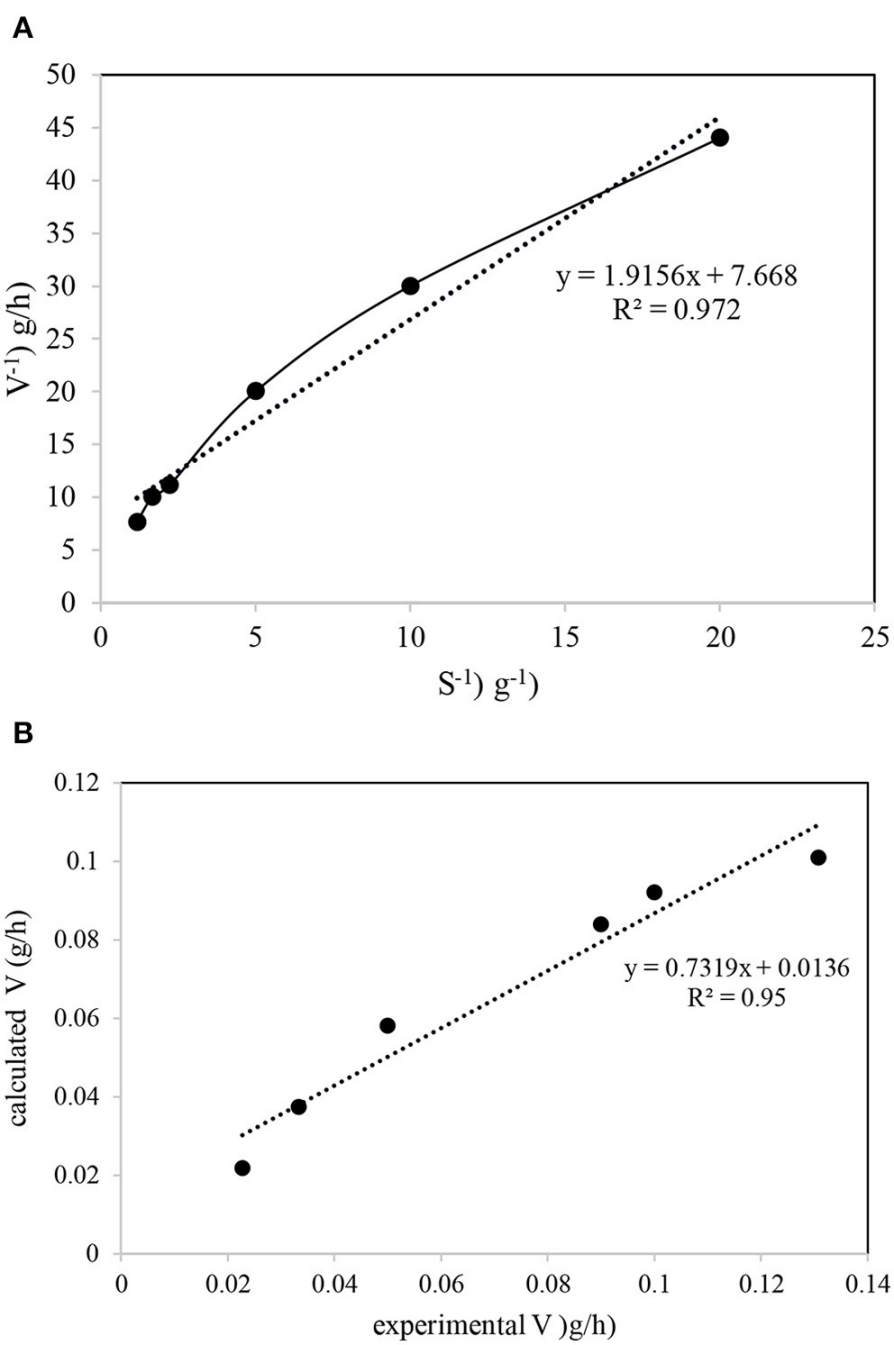
Michaelis-Menten plot for determination of kinetic coefficients in the system: **(A)** V^−1^ and S^−1^ plotting; **(B)** experimental and calculated data on one plot.

The data required for the Michaelis–Menten model was obtained from reviewing previous work that used bioelectrical denitrification in the presence of ibuprofen as the organic carbon source in the experimental setup. The detailed results for nitrate and ibuprofen removal are reported elsewhere (Hoseinzadeh et al., 2017c, 2018). The half-saturation concentration (*K*_*s*_) and the maximum specific utilization rate (*V*_*max*_) of nitrate in the development process was determined based on the data obtained from a nitrate shock-study stage (removal of more than 99% in feed wastewater). The *V*_*max*_ and *K*_*s*_ values were determined from the intercept and the slope, respectively, of the plot of 1/*V* vs. 1/*S* (Figure 6). The linear plot of 1/*V* vs. 1/*S* indicates a high degree of fitness (*R*^2^ = 0.972), as did the plot of *V* obtained from the experimental data against the Michaelis-Menten model (*R*^2^ = 0.95) (Figure 6B). Based on the equation fitted to the experimental data, *V*_*max*_ and *K*_*s*_ were determined to be 3.13 g nitrate/d and 5.99 g nitrate/L, respectively. A *V*_*max*_ value of 0.907 g nitrate/d was reported for the nitrate removal from continuous stirred tank reactors using the bacterium *Azospira sp*. OGA 24 (Rossi et al., 2014), while nitrate removal by *Klebsiella* sp. FC61 in the presence of Fe^3+^ only displayed a nitrate removal in the range of 0.005–0.019 g nitrate/d (Su et al., 2016). The maximum nitrate removal rate for the combined aerobic biological phenol and batch-suspended growth cultures was reported as 0.025 g nitrate/d (Vasiliadou et al., 2008). De Filippis et al. (2013) obtained a maximum nitrate removal rate in activated sludge reactors (ASRs) with a high nitrate concentration (>3 g/L) in a range of 0.044–0.333 g nitrate/L·d. This range is 10 times lower than that in the present study (3.13 g nitrate/d). The Michaelis-Menten model results for our study confirm that the performance of ACBER in ibuprofen removal and denitrification was not inhibited by the ibuprofen concentration at the studied conditions. Indeed, the ibuprofen concentration as a hard biodegradable organic matter (Hoseinzadeh et al., 2016) at which the ibuprofen removal could be inhibited in the ACBER as high as 1,000 mg/L. The higher *V*_*max*_ value obtained in the present study, compared to previous reports, suggests that the nitrate removal ability of our system is much greater than that of conventional biological processes. More specifically, a shorter reaction time, and, thus, a smaller reactor size, is required for treating high-level nitrate wastewater when using the hard-biodegradable organic carbon source employed in this system, compared with a conventional bioprocess such as an ASR. The incorporation of the electric field into the bioreactor in the Monod equation, with modifications by Zeyoudi et al. (2015), was used for the biokinetics process study. Using Equation 5, μ values of 0.84 ± 0.025 and 0.397 ± 0.012 h^−1^ were reported for biofilms formed on SS304 and CC electrodes, respectively. In addition, μ_*m*_ for the biofilms formed on SS304 and CC electrodes were 1.08 ± 0.03 and 0.51 ± 0.015 h^−1^, respectively. These results correspond to a doubling time of 0.82 ± 0.024 h and 1.74 ± 0.052 h for the biofilms formed on the SS304 and CC electrodes, respectively. Zeyoudi et al. (2015), showed μ values in the range of 0.02–0.08 h^−1^, 10 times lower than that obtained from the system in the present study. Furthermore, the doubling time for their data was 10–40 and 6–20x lower than that obtained in the present study when compared with the biofilms formed on the SS304 and CC electrodes, respectively. It should be noted that the comparison to the publication can be misleading as the applied current is different (AC instead of DC), but it was the most similar study to that presented.

#### Dehydrogenase Activity (DHA)

**Figure 7:**
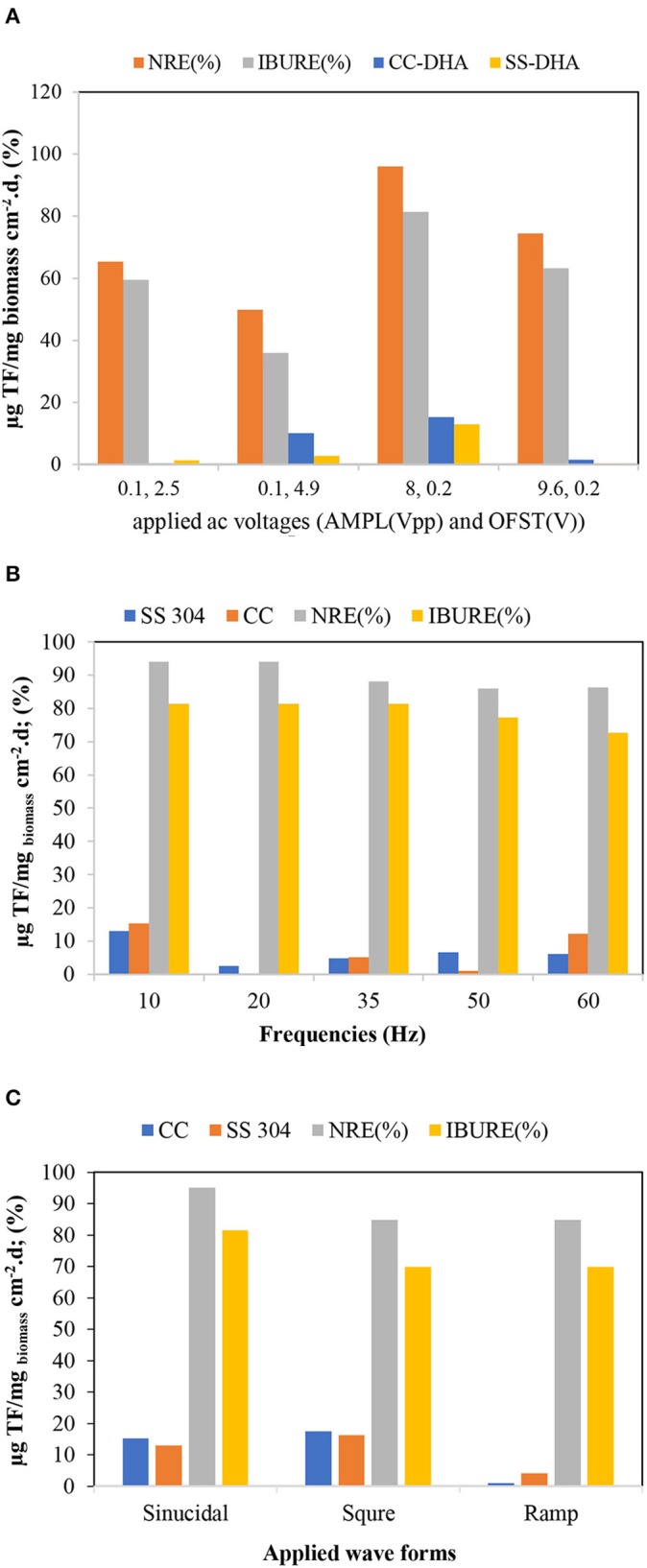
Dehydrogenase activity (DHA) determination for the system biofilm: **(A)** AC magnitude; **(B)** AC frequency (sin. waveform and 8 Vpp); **(C)** AC waveform (8 Vpp and 10 Hz).

**Figure 8:**
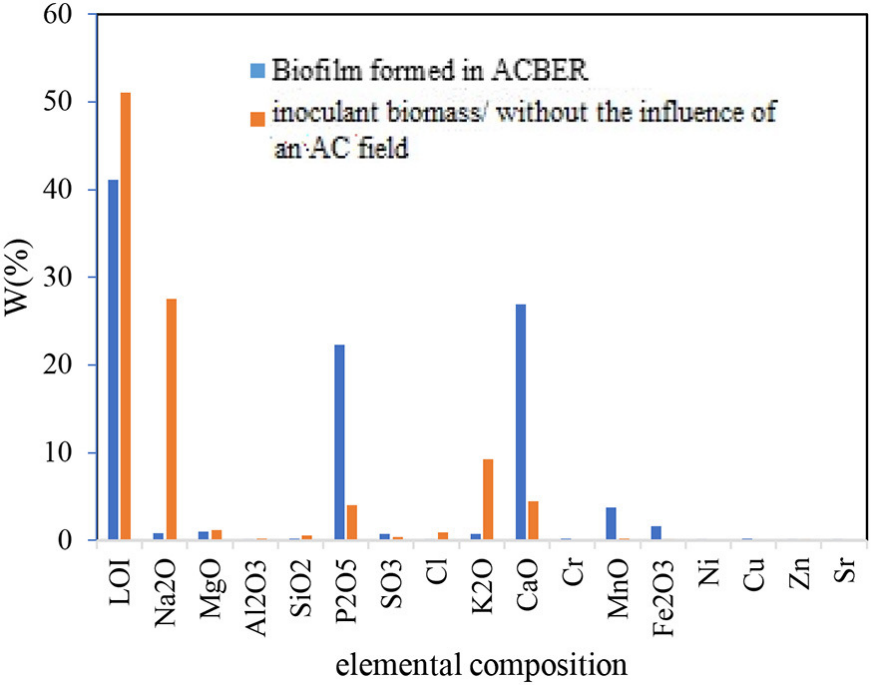
XRF results from the inoculation mass and formed biofilm.

An important concern related to the application of the AC was little or no activity on the biofilms. In this study, DHA was used as an index of microbiological activity. In general, the maximum biofilm DHA formed on the CC and SS304 electrodes because of the AC voltages, frequencies, and waveform to the ACBER. The DHA results for the biofilms on the CC electrode were higher than those for the biofilms on the SS304 electrode. As Figure 7 shows, the greatest DHA was obtained at 8 Vpp. In addition, with voltage set at 8 Vpp and 0.2 V, the DHA and pollutant removal capacity was based on the AC frequencies and waveforms. Using AC (energetic and non-thermal) in the applied range compressed and transferred the electrical energy to the bacteria due to discharged electricity (Hoseinzadeh and Rezaee, 2015). However, at this voltage, the transfer caused the decomposition of target pollutants. Furthermore, there was no notable correlation between the efficiency of pollutant removal and DHA, which differs from the conventional concept that higher enzyme activity results in a lower quantity of organics in the effluent. As shown in Figure 7, the DHA content varied under different applied voltages. The highest DHA was obtained at 8 Vpp. In addition, the results demonstrate that suitable electro-stimulation has a positive effect on bacteria by promoting their metabolism and activity (You et al., 2015; Liu et al., 2016). The frequency and waveform are two additional important properties for AC. Subsequently, the impact of both on the DHA was assessed. As Figure 7 shows, the highest DHA was obtained at 10 Hz with a square waveform. Hence, this finding suggests that altering the applied voltage, frequency, and waveform of AC to the bioreactor can increase or decrease the DHA. We used X-ray fluorescence (XRF) analysis to determine the biofilm elemental content. As the XRF analysis shows, the Ca^+2^ within the biofilm increased (Figure 8). We propose that the increase of Ca^+2^ is the result of the effect of AC on biofilm (Beebe, 2015); however, this requires further studying. Notably, the XRF analysis of the biofilm vs. the inoculation mass reveals a higher phosphate content of the biofilm mass. Applying AC may lead to conformational changes within the phosphorylated protein (Kobir et al., 2011). Therefore, phosphorylation/dephosphorylation is an important physiological change to assess. By this mechanism, it is probable that phosphates are transferred from ATP to the substrate. In addition, the ratio of volatile suspended solids to the total suspended solids decreased from 82 to 53.7% for the biofilm, while the loss-on-ignition also decreased from 52.1 to 41.12%. These results support our proposal that bacteria inoculated in the system are vulnerable to AC electricity.

#### Apoptosis Assay by Flow Cytometry Analysis

**Figure 9:**
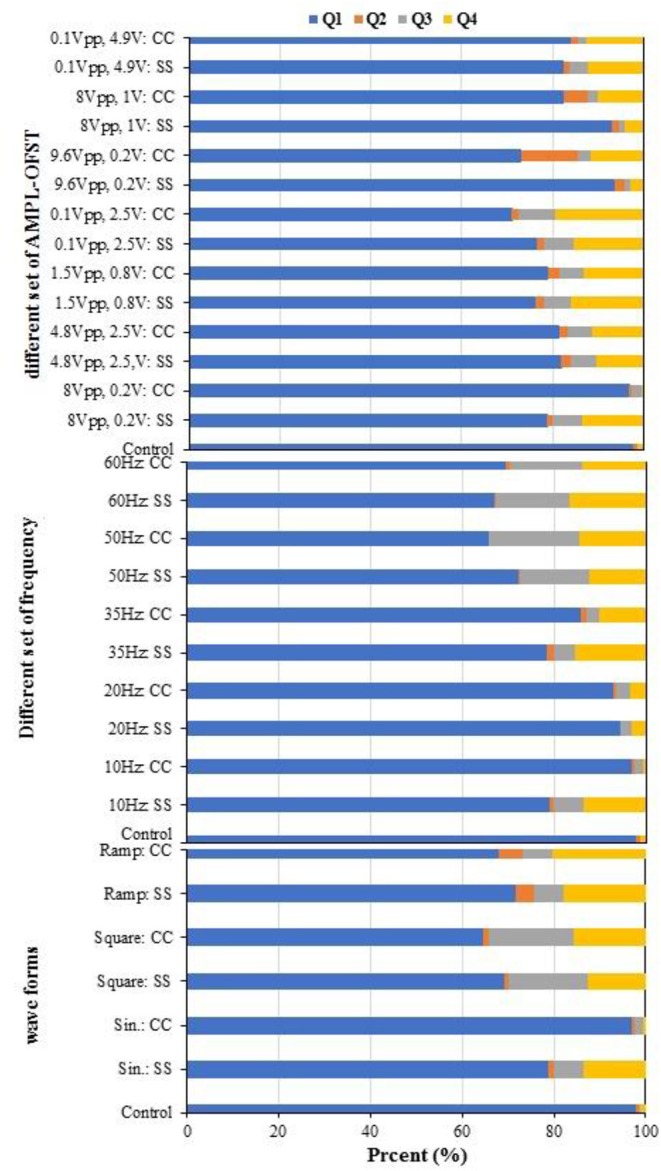
Biofilm viability assessed with Flow cytometry utilizing FITC/PI staining [Studying effect of AMPL-OFST, Studying effect of frequency effect and studying effect of waveform; SS, biofilm formed on stainless steel electrodes; CC, biofilm formed on carbon cloth (CC) electrodes].

Double staining with Annexin V-FITC/PI was utilized for a cytometry analysis to demonstrate the biofilm viability under the influence of different AC voltages, frequencies, and waveforms. Cells were gated by plotting FSC-A vs. SSC-A to identify any changes in the scatter properties of the cells. The Annexin V-FITC-A vs. propidium iodide-A plots show the populations corresponding to the viable and non-apoptotic (Annexin V-PI−), as well as early (Annexin V+PI–) and late (Annexin V+PI+) apoptotic cells. As Figure 9 shows, in the without the influence of an AC field (control) samples, the majority of cells (97.71%) were viable and non-apoptotic (Annexin V-PI), while the AC amplitude in the utilized range caused an increase in the apoptotic and necrotic cells. The number of apoptotic cells differed between the SS304 and CC electrodes at the studied AC amplitudes. This may be due to the different electrode material behaviors at varying AC amplitudes. The lowest populations of early apoptotic cells (Annexin V+PI−) occurred at 8 Vpp and 0.2 V with the CC electrode (0.51 vs. 0.9% for the CC electrode and control sample, respectively). By changing the AC amplitude, a slight increase in the Annexin V+PI+ population was observed, which indicates late apoptotic or dead cells. Any impact on the external or internal membranes of the cell can profoundly affect its performance, thus, can cause cell death (Harnisch et al., 2011). Generally, the number of cells entering apoptosis was decreased by supplying an AC with an amplitude of 8 Vpp, supporting the hypothesis of an anti-apoptotic or low apoptotic effect of AC at studied voltages. As previously established, cell shrinkage and internal membrane destabilization occurs when the AC induces biofilm formation during apoptosis, which causes the release of important intracellular mediators of cell signaling or second messengers, such as calcium (Beebe, 2015). In this context, a Ca^+2^ increase in the biofilm is shown by the XRF. In the AC frequency effect study, the results (Figure 9) shows that the lowest frequency of late apoptotic cells was 20 Hz for both electrodes (3.14 and 3.45% for the SS304 and CC electrodes, respectively), while the late apoptotic cells for the other frequencies were in the same range. The non-apoptotic cell population decreased with increasing frequency, progressing from 95.59 ± 1.86 to 68.28 ± 1.86% for 10 and 60 Hz, respectively. Whereas, the application of lower frequency pulses resulted in differing display times (s) and AC voltage or current waveforms, compared to those displayed at a higher frequency (360 vs. 60 s for 10 and 60 Hz, respectively). As the use of lower frequencies provided the advantage of lower losses (Hoseinzadeh and Rezaee, 2015), which are proportional to frequency, operation at 10 Hz can lead to operation below the required energy. In addition to the application of the AC voltage to the biofilm bacteria, the ibuprofen, used as the organic carbon source, can result in decreased bacterial viability. In a study, Wang and Gunsch (2011) proved that ketoprofen, naproxen, carbamazepine, and gemfibrozil byproducts have an impact on bacterial membrane integrity, which leads to decreased cell viability. The non-apoptotic population was decreased by changing from sine waves to ramp and square waves (87.89, 69.8, and 66.8%, respectively). Moreover, the lowest late apoptotic cell frequency was for sine waves with the CC electrode (Figure 9). The sine wave is the closest waveform to AC behavior (Hoseinzadeh and Rezaee, 2015; Hoseinzadeh et al., 2017b,c, 2018), and is unaffected by an electric circuit comprising a resistor, inductor, and capacitor. AC generation results in the sine wave and shape being unaffected by the components. Therefore, the sine wave form is favorable.

## Conclusion

Based on the results, it was expected that the induced changes in surface properties and cell shape would contribute to the electro-kinetic movement of bacteria on the subsurface. However, exposure to an AC could cause changes in bacterial cell properties such as increases in activity and the predominance of specific species that lead to different inoculant biomass (without the influence of an AC field) populations instead of the formation of biofilm on the electrodes. Such changes could stimulate the attachment of the bacteria to solid surfaces and, therefore, diminish the reversibility ratio. The AC at the studied voltage range of 8 Vpp amplitude has a stimulating effect because the highest DHA was obtained at this voltage. In addition, the flow cytometry analysis showed a relatively low number of apoptotic cells; thus, it can be concluded that a low voltage-low frequency AC induces significant changes in bacterial metabolic activity, but causes no significant change in their viability.

## Data Availability Statement

All datasets generated for this study are included in the article/supplementary material.

## Author Contributions

EH did conception and design, acquisition of data, or analysis and interpretation of data, and writing. AR did conception and design, analysis and interpretation of data, and drafting the article or revising it critically for important intellectual content. MF and CW did revising it critically for important intellectual content and final approval of the version to be published.

### Conflict of Interest

The authors declare that the research was conducted in the absence of any commercial or financial relationships that could be construed as a potential conflict of interest.
